# Analysis of tumor infiltrating CD4+ and CD8+ CDR3 sequences reveals shared features putatively associated to the anti-tumor immune response

**DOI:** 10.3389/fimmu.2023.1227766

**Published:** 2023-08-04

**Authors:** Andrea Aran, Gonzalo Lázaro, Vicente Marco, Elisa Molina, Ferran Abancó, Vicente Peg, María Gión, Laia Garrigós, José Pérez-García, Javier Cortés, Mercè Martí

**Affiliations:** ^1^ Immunology Unit, Department of Cell Biology, Physiology, and Immunology, Institut de Biotecnologia i Biomedicina (IBB), Universitat Autònoma de Barcelona (UAB), Bellaterra, Spain; ^2^ Pathology, Hospital Quironsalud Barcelona, Barcelona, Spain; ^3^ Pathology Department, Vall d’Hebron University Hospital, Barcelona, Spain; ^4^ Department of Morphological Sciences, Universidad Autónoma de Barcelona, Bellaterra, Spain; ^5^ Spanish Biomedical Research Network Centre in Oncology (CIBERONC), Madrid, Spain; ^6^ Medical Oncology Department, Ramón y Cajal University Hospital, Madrid, Spain; ^7^ International Breast Cancer Center (IBCC), Pangaea Oncology, Quironsalud Group, Barcelona, Spain; ^8^ Medical Scientia Innovation Research (MedSIR), Barcelona, Spain; ^9^ Department of Medicine, Faculty of Biomedical and Health Sciences, Universidad Europea de Madrid, Madrid, Spain; ^10^ Biosensing and Bioanalysis Group, Institute of Biotechnology and Biomedicine, Universitat Autònoma de Barcelona, Bellaterra, Spain

**Keywords:** tumor-infiltrating lymphocytes, CD4+ T cells, CD8+ T cells, T cell receptor, breast cancer

## Abstract

**Introduction:**

Tumor-infiltrating lymphocytes (TILs) have predictive and prognostic value in breast cancer (BC) and exert a protective function against tumor growth, indicating that it is susceptible to treatment using adoptive cell transfer of TILs or T cell receptor (TCR)-based therapies. TCR can be used to identify naturally tumor-reactive T cells, but little is known about the differences in the TCR repertoires of CD4+ and CD8+ TILs.

**Methods:**

TCR high-throughput sequencing was performed using TILs derived from the initial cultures of 11 BC biopsies and expanded and sorted CD4+ and CD8+ TILs as well as using PBMCs from healthy donors expanded and sorted using the same methodology.

**Results:**

Physicochemical TCR differences between T cell subsets were observed, as CD4+ TILs presented larger N(D)Nnt TRB sequences and with a higher usage of positively charged residues, although only the latest was also observed in peripheral T cells from healthy individuals. Moreover, in CD4+ TILs, a more restricted TCR repertoire with a higher abundance of similar sequences containing certain amino acid motifs was observed.

**Discussion:**

Some differences between CD4+ and CD8+ TCRs were intrinsic to T cell subsets as can also be observed in peripheral T cells from healthy individuals, while other were only found in TILs samples and therefore may be tumor-driven. Notably, the higher similarity among CD4+ TCRs suggests a higher TCR promiscuity in this subset.

## 1 Introduction

The study of the antitumoral immune response has become a research focus and has led to an increasing number of therapies based on activating, enhancing, or modulating different components of the immune system, known as immunotherapies (1, 2), such as adoptive cell therapy using tumor-infiltrating lymphocytes (TILs), chimeric antigen receptor T cells (CAR-T cells), and immune checkpoint inhibitors (ICIs). Recently, the use of immunotherapies has demonstrated effectiveness in breast cancer (BC), the most frequent cancer in women and the most prevalent worldwide, assuming a great advance for certain types of aggressive tumors and/or those for whom there is a need to find alternative therapies, such as triple-negative BC (TNBC) or metastatic BC (3–5).

Tumors (including BC) with heavy immune cell infiltration or hot tumors have better prognoses than those with low immune cell infiltration or cold tumors. However, TILs are not present in all individuals, and TIL can also acquire a state of dysfunction or exhaustion, thereby losing effector functions (6). Therefore, the degree of infiltration by itself is not always indicative of an effective anti-tumor immune response; the functional state of T cell function is also important. In fact the CD8+/regulatory T cell (Treg) ratio is a good prognostic biomarker (7), indicating that that an efficient antitumor response depends on the presence of infiltration by active T cells not subjected to the inhibitory action of Tregs. Even so, a good outcome is not always ensured, as other mechanisms, such as TILs inhibition by suppressor signals in the tumor microenvironment, may have a critical influence. Nonetheless, TILs are a natural source of antitumor materials, and more recently, therapeutic approaches have focused their attention on the T cell receptors (TCRs) of TILs, developing therapeutic products with engineered transgenic TCRs that might exhibit improved recognition of cancer cells (8–10).

The antitumor response requires the activation of both CD4+ T cells, which are crucial for orchestrating and sustaining the initial response and generating immunological memory, and CD8+ T cells, which are crucial for their capacity to directly recognize and kill tumor cells. Both T cell subsets require recognition of antigen-HLA complexes through their TCR to initiate T cell activation; that is, CD4+ and CD8+ T cells recognize HLA class II (HLA-II) and HLA class I (HLA-I)-associated peptides, respectively. The TCR repertoire of different subsets is determined by three different factors: (i) the differences in the peptides presented by HLA-I and HLA-II molecules, which may contribute to the selection of different CD4+ and CD8+ TCRs with different physicochemical properties during thymic selection, (ii) the antigen availability influenced by a differential presentation; that is, class I peptides can be directly presented by tumor cells and recognized by CD8+ T cells, whereas class II peptides must be captured, processed, and presented by APCs (dendritic cells, B cells, and macrophages); therefore, a constant release of tumor antigens in high amounts from dead or apoptotic tumor cells is necessary to maintain a class II response, and (iii) the HLA expressed genotype.

Previous studies have described intrinsic dissimilarities between peripheral CD4+ and CD8+ TCRs although these differences have not been assessed for TILs TCRs. Most TCR studies on cancer have focused on TCR repertoire features, as the TCR provides the antigen specificity of T cells, representing a biomarker that allows to analyze the status and evolution of TILs. TCR diversity analysis is one of the most evaluated features, used to monitor the presence of clonal expansions, indicating an activation and response of T cells, but other features such as the convergence level, or convergent recombination (CR), which represents the number of CDR3nt sequences encoding a specific number of CDR3aa, can also suggest a T cell response. Different T cells with the same CDR3aa but with different origins (different CDR3nt) may denote tumor recognition and higher CR levels among public TCRs (shared among different individuals) have been reported in breast cancer (BC) (11). The use of various TCR repertoire features to predict prognosis and response to ICI therapies has also been evaluated by several groups and recently reviewed by ours (12, 13), but little is known about the differences in these properties between TILs subsets. The identification of specific TCRs is mandatory for designing TCR-based therapies, and both CD4+ and CD8+-specific TCRs have been successfully used for treatment (14, 15). Therefore, understanding how TCRs are configured in different T cell subsets and knowing which factors in the repertoire can indicate activation and/or response processes, as well as whether these have similar or different behaviors in different subpopulations in the tumor microenvironment, may help us select better TCRs candidates to improve the development of personalized therapies.

Considering all this, the objective of this study was to analyze differences in CD4+ and CD8+ TILs in BC, focusing both on their TCR properties as well as on the repertoire features that may point out a different response. We have studied 11 BC biopsies with different etiology and have performed TCR HTS both in initial TILs cultures (from small biopsy-slices cultured as explants in the presence of low IL-2 doses) and in expanded and sorted CD4+ and CD8+ TILs. Our data indicate that TCRs from CD4+ and CD8+ T cells present some physicochemical differences, some of which are attributable to the cellular subtype while others are only observed in the tumoral infiltrates. Not only differences in the TCR diversity were observed but also in the sequence similarity, i.e., CD4+ TILs show a more restricted repertoire and frequently present conserved aminoacidic motifs in the TRB CDR3 sequences, both intra- and interindividual, while CD8+ TILs are more diverse and exhibit more variable TRB CDR3 sequences.

## 2 Material and methods

### 2.1 Breast cancer TILs samples

Four triple-negative, three luminal A, and four luminal B breast cancer biopsies from 11 female patients, with age raging from 42 to 74 at the time of the diagnosis used in this study are summarized in **Supplementary Table 1**. Biopsies were cut into small slices covering the entire tissue sample to maintain representation of TILs at different locations in the surgical sample. Some slices were frozen in liquid nitrogen, whereas others were cultured as explants in 48-well plates (biopsy-slices used for TCR analysis are summarized in **Supplementary Table 1**). Biopsy-derived TILs, were cultured in 1 ml of complete culture T cell medium (IMDM GlutaMAX™ (Gibco) + 10% decomplemented human serum + antibiotic/anti-mycotic (Sigma)) using low levels of IL-2 (100U/ml, provided by the NIH). Half of the medium with the corresponding IL-2 was renewed every 5 days.

**Figure 1:**
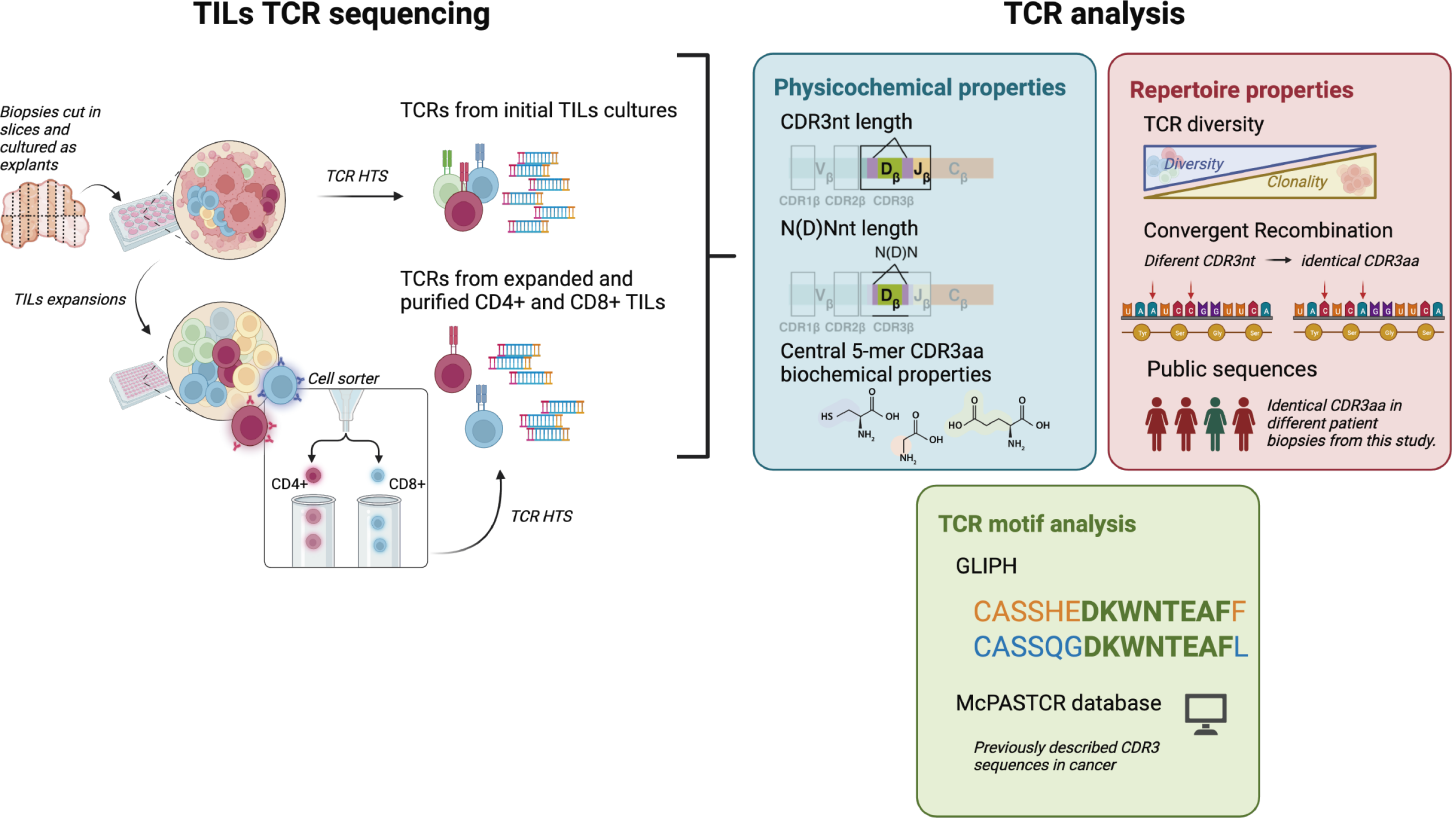
CD4+ and CD8+ TILs TCR repertoire analysis. Biopsies were cut in several slices cultured as explants and TILs from the initial cultures were harvested after 7-15 days for both TCR HTS and T cell expansions. After 15 days, expanded CD4+ and CD8+ TILs were purified using a cell sorter and TCR HTS was performed. We analyzed TCR physicochemical properties (CDR3nt and N(D)Nnt length and biochemical properties of the central 5-mer of the CDR3aa), TCR repertoire properties (diversity, convergent recombination, and the presence of public sequences), and the presence of motifs in the CDR3 sequences. Clustered sequences obtained from the motif analysis (GLIPH2 (16, 17)) were compared with the literature (using McPAS-TCR (18)). Created with Biorender.com.

After approximately 10 days, biopsy-derived TILs (referred to as TILs-initial) were harvested and analyzed to assess the abundance of CD4+ and CD8+ TILs by flow cytometry and cell pellets were collected simultaneously for TCR analysis (**Figure 1**). Between 2-4 weeks after the initial culture, some TILs were harvested, expanded, and sorted (referred to as TILs-CD4+ and TILs-CD8+) for TCR analysis.

### 2.2 Peripheral T cell samples from healthy donors

Peripheral T cells, used as controls, were obtained from 20 ml of blood from six healthy donors: three men and three women ranging in age from 20 to 30 years old (summarized in **Supplementary Table 2**). We considered only healthy donors without any type of infection at the time of blood extraction or without any disease with an immune etiology, and excluded donors recently vaccinated. Blood was extracted from the Autonomous University of Barcelona Health Care Service and consent was obtained from all volunteers. PBMC were obtained from the peripheral blood, diluted 1:2 in PBS, and separated using a Ficoll-Paque density gradient (Lymphoprep; Alere Technologies). From PBMCs collected from the phase, a portion of cells was directly used for TCR analysis using bulk cells as well as sorted CD4+ and CD8+ cells (referred to as PBMCs, P-CD4+ and P-CD8+, respectively). The remaining was cultured to perform TCR analysis after T cell expansions. Expanded cells were used in bulk as well sorted CD4+ and CD8+ cells (referred to as ePBMCs, eP-CD4+ and eP-CD8+, respectively)

### 2.3 T cell cultures and expansions

Between 2-4 weeks after the initial TILs cultures, cells were used for T cell expansion using the T cell TransAct™ (MiltenyiBiotec). T cells were plated in 96-well plates at 3 × 10^5^ cells/well in 200 μL complete T cell medium. On the second day, according to the manufacturer’s instructions, the reagent was removed and replaced with 180 μl of fresh T cell complete medium, and cell suspensions were split every 2 days (1:2 ratio). After a total of 14 days, cells were harvested for FACS sorting and TCR analysis. PBMCs from healthy donors cultured directly after obtention were expanded using the same procedure.

### 2.4 Flow cytometry and cell sorter

Between 2-5 x 10^5^ cells were stained with anti-human antibodies for 20 min at 4°C in the dark in PBS + 2% fetal bovine serum (FBS). After incubation, the cells were washed and analyzed by flow cytometry. Antibodies used for flow cytometry were FITC-conjugated anti-human CD3 (RRID : AB 10893003), PE-conjugated anti-human CD4 (RRID : AB_393790), and APC-conjugated anti-human CD8 (RRID : AB_10642579) (BD Pharmingen). A BD FACS Canto flow cytometer was used for analysis. Analyses were performed using the FlowJo, RRID : SCR_008520, and FACS Diva software. The same staining procedure and antibodies were used for the CD4+ and CD8+ T cell sorting, harvested after 14-15 days of expansion method. Cell sorter used was a BD FACsJazz. Cells collected from the sorter were pelleted and used for the TCR analysis.

### 2.5 TCR library preparation

1 × 10^5^ - 1 × 10^6^ cells were used for RNA extraction, isolated using the RNeasy Micro Kit (Qiagen) with on-column DNase digestion using an RNase-free DNase set (Qiagen) following the manufacturer’s instructions. The amount and integrity of RNA were measured externally by the Genomics Core Facility of the University Pompeu Fabra (UPF) using an Agilent 2100 Bioanalyzer (Agilent Technologies) and RNA 6000 Nano or RNA 6000 Pico chips. Samples with RIN <7 were excluded. TCR profiling was performed using a SMARTer Human TCR a/b Profiling Kit (Takara Bio, Shiga, Japan). TRA and TRB sequencing was applied both to all TILs samples, while only TRB sequencing was applied to PBMCs from healthy donors’ samples. Library purification was performed using Agencourt AMPure XP Beads (Beckman Coulter) and libraries were analyzed and validated on an Agilent 2100 Bioanalyzer using a DNA 1000 kit (Agilent Technologies). HTS was performed on an Illumina MiSeq sequencer using a 600-cycle MiSeq Reagent Kit with paired-end 2 × 300 bp reads.

### 2.6 TCR repertoire analysis

Raw TCR sequencing data were aligned using MiXCR Immune Repertoire Analyzer (19) (RRID : SCR_018725) and processed using VDJTools (20). Non-productive clonotypes were excluded and routine decontamination was performed to eliminate cross-sample contamination. Before the length, biochemical properties and motifs analysis, pre-processed data were collapsed by CDR3aa sequences using VDJTools (20), that is, clonotypes with different nucleotide sequences encoding the same CDR3aa sequence were summed, and frequencies were recalculated. TCR data from the different biopsy-slices from the same patient were merged for the comparative analyses except when indicated. The length, biochemical properties, diversity, CR level and presence of public CDR3aa sequences were also analyzed using VDJTools^2^ functions.

Motif analysis was performed using the pooled CDR3aa TRB sequences in the CD4+ and CD8+ sets using the GLIPH2 (16, 17) tool. Only those motifs with a Fisher score >0.5 and an expansion score >0.5 were used to select only those motifs that were significantly enriched. The TCR sequences with a frequency > 0.1%, the public TCR sequences, and the TCR sequences obtained from the GLIPH2 (16, 17) analysis were compared with the McPAS-TCR (18) database with a Levenshtein distance of 1.

### 2.7 Statistical analysis and figures

Statistical analyses, as well as data normalizations, and figures were generated using GraphPad Prism, RRID : SCR_002798 (version 7.0). Pearson r correlations were used to assess statistical significance in the study of the relationship between CD4+ and CD8+ percentages in TILs-initial samples and the TCR diversity, as well as in the study of the relationships between the number of reads and CDR3 sequences obtained in TILs-CD4+ and TILs-CD8+ samples with the number of motifs obtained by GLIPH2 (16, 17). Ordinary one-way ANOVA with Tukey’s multiple comparison tests were used to compare CDR3 properties in different TILs and PBMCs samples. Paired t-tests were used to analyze differences in diversity in CD4+ and CD8+ samples from the same biopsy sections. Ordinary two-way ANOVA with Sidak’s multiple comparison tests were used to assess statistical significance between samples before and after *in vitro* expansions in bulk PBMCs and purified CD4+, and CD8+ T cells. The results of the statistical tests are shown in figures. UpSet plots were generated with R3 (version 3.6.1) using the “dplyr” (21), “RColorBrewer” (22), and “UpsetR” (23) packages.

## 3 Results

### 3.1 Analysis of CDR3 differences between CD4+ and CD8+ TILs and peripheral T cells from healthy donors

We analyzed the CDR3nt and N(D)Nnt regions sequence lengths and the biochemical properties of the CDR3aa sequences in CD4+ and CD8+ TILs. Unlike peripheral T cells, TILs are supposed to be activated in the draining lymph nodes and then migrate to the tumor site. Therefore, TCR differences may be attributed to the differential selection of certain clones, probably tumor antigen selected clones. Moreover, to rule out the possibility that findings were instead due to a bias in T cell rate of growth during the *in vitro* expansion, we analyzed TRB transcripts from healthy peripheral T cells before and after being subjected to the same protocol expansion than the BC TILs as a control. These samples were also used as controls to assess whether TCR differences could be observed in healthy peripheral T cells. As analyses from TRA sequencing did not show important remarks, we only performed TRB HTS on healthy donors’ T cells to compare with results from TILs samples. TRA derived data and analysis from TILs can be found at **Supplementary Data** and **Supplementary Figures 1, 4, 6, 7**.

From TILs derived after the initial short culture with IL-2 (from now on, TILs-initial samples), we obtained a total number of 35357 TRB CDR3nt sequences (**Supplementary Table 1**). After TILs expansions using anti-CD3/anti-CD28 agonists and subsequent CD4/CD8 sorting (from now on referred to as TILs-CD4+ and TILs-CD8+ samples, respectively), we obtained 9245, and 5568 TRB CDR3nt sequences respectively (**Supplementary Table 1**). The number of CDR3nt sequences obtained in TILs samples varied widely and could not be associated with the global infiltration level of TILs, except for the BTLQ7 which was highly infiltrated and from which we obtained the highest number of sequences. Therefore, this variation could be related with the different degrees of infiltration levels in a single biopsy.

The TRB HTS was performed using peripheral T cells from healthy donors, both using bulk samples and CD4+ and CD8+ sorted cells, before and after *in vitro* expansions (from now on, PBMCs, P-CD4+ and P-CD8+ for samples obtained before expansions, and ePBMCs, eP-CD4+ and eP-CD8+ for samples obtained after expansions). As expected, we obtained much more sequences using peripheral T cells, that is 49336, 362524 and 220912 TRB CDR3nt sequences from PBMCs, P-CD4+ and P-CD8+ samples, respectively, and 143376, 256681 and 179252 TRB CDR3nt sequences from ePBMCs, eP-CD4+ and eP-CD8+ samples, respectively (**Supplementary Table 2**).

**Figure 2:**
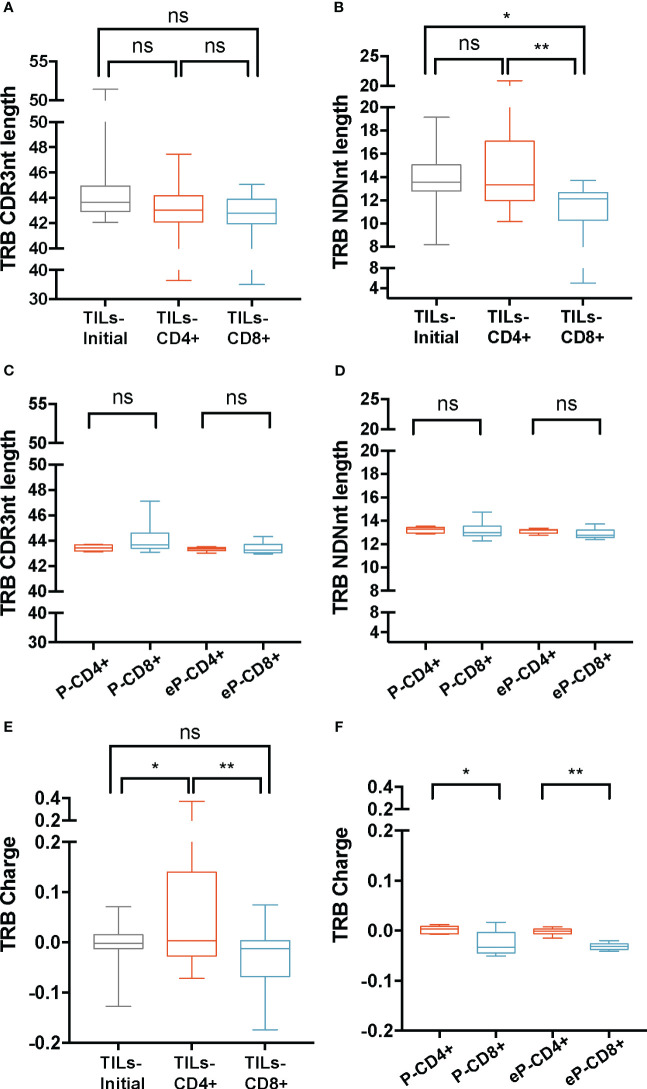
TRB physicochemical properties in TILs and peripheral T cells from healthy donors. CDR3nt length and NDNnt length analysis of TRB CDR3nt sequences in TILs samples **(A, B)**, respectively) and PBMCs from healthy donors **(C, D)**, respectively). TILs-initial and TILs-CD4+ samples presented significantly larger TRB NDNnt sequences compared with TILs-CD8+ samples. This difference was not observed in the PBMCs samples. The charge level of TRB CDR3 sequences in TILs **(E)** and PBMCs **(F)** revealed a significantly higher usage of positively charged residues in TILs-CD4+ samples compared to TILs-initial and TILs-CD8+ samples. This difference was also observed in CD4+ samples from PBMCs, both before (P-CD4+ vs. P-CD8+) and after (eP-CD4+ vs. eP-CD8+) expansions. TILs samples derived from BC biopsies and control samples used are summarized in **Supplementary Tables 1** and **2**, respectively. Values from each sample are available in **Supplementary Data**. ns (not significant), p >0.05; *, p ≤ 0.05; **, p ≤ 0.01.

TRB CDR3nt mean length ± SD was 44 ± 1.9 on TILs-initial samples and 43 ± 2.4 on both TILs-CD4+ and TILs-CD8+ samples (**Figure 2A**). We checked whether the variability in the CDR3nt length was caused by differences in the N(D)Nnt region and found that the TRB N(D)Nnt mean length of TILs-CD8+ samples (11.4 nt ± 2.2) was significantly shorter than that of TILs-CD4+ samples (14.4 ± 3.1 nt, p=0.004), and of TILs-initial samples (13.8 ± 2.1 nt, p=0.019) (**Figure 2B**). In control samples, we did not find significant differences in the TRB N(D)Nnt sequence length neither of P-CD4+ and P-CD8+ samples nor of eP-CD4+ and eP-CD8+ samples (**Figures 2C, D**).

Subsequently, the biochemical properties (hydropathy index, charge, and polarity) of the central 5aa of the CDR3 beta chains were analyzed (**Figures 2E, F**; **Supplementary Figure 2**). Data were weighted by the frequency of sequences and normalized to subregion size (5aa). Among the three properties analyzed, we only observed differences in the charge of the TRB sequences, specifically, the TILs-CD4+ samples presented the highest mean value (0.06 ± 0.12), which was significantly different from that observed in the TILs-CD8+ (-0.04 ± 0.07, p=0.005) and the TILs-initial samples (-0.01 ± 0.04, p=0.045) (**Figure 2E**). This indicated an increased presence of positively charged amino acids in the central 5-mer of the CDR3 beta chain in TILs-CD4+. In the control analysis, we found a significant difference in the charge, with an average value of 0 ± 0.008 in P-CD4+ and of -0.03 ± 0.02 in the P-CD8+ (p=0.012, **Figure 2F**) and a statistically significant increase was observed after expansions (p=0.008). Although this may suggest that expansions can slightly modify the repertoire, none of the properties analyzed showed significant differences after expansion (**Supplementary Figures 3A-E**). Thus, we concluded that the TRB N(D)Nnt length difference was a feature from TILs, whereas the difference in the usage of positively or negatively charged amino acids in the central 5-mer of CDR3aa was a CD4+ and CD8+ intrinsic TCR feature.

### 3.2 Diversity analysis of CD4+ TCR from TILs reveals a restricted repertoire

The heterogeneity of T cell subsets may extend beyond the physicochemical properties of the TCR. By studying other TCR repertoire features, such as diversity, the TCR can be considered as a biomarker of response, as diversity is associated with the presence of clonal expansions. Moreover, the TCR diversity can vary among different T cell subsets, reflecting the differences in antigen recognition capabilities between CD4+ and CD8+ T cells. Hence, in this study, we investigated the TCR diversity in TILs-CD4+ and TILs-CD8+ samples. TCR data were stratified both by patients and by the different biopsy-slices cultured. The normalized Shannon-Wiener index (nS-W) was used to compare TILs-initial with TILs-CD4+ and TILs-CD8+ TCR diversities.

**Figure 3:**
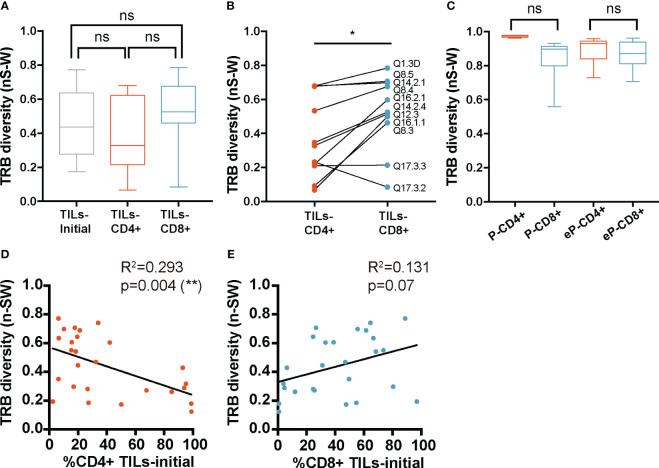
TRB diversity in TILs and peripheral T cells from healthy donors. **(A)** TRB diversity analysis calculated using all samples did not show differences between groups. **(B)** The paired analysis of TRB diversity calculated using only the values from slices from which TILs-CD4+ and TILs-CD8+ samples were available revealed a significant higher diversity in the CD8+ group. **(C)** TRB diversity levels were not significantly different between CD4+ and CD8+ T cells from PBMCs neither before (P-CD4+ vs. P-CD8+) not after (eP-CD4+ vs. eP-CD8+) *in vitro* expansions. The TRB diversity inversely correlated with the percentage of CD4+ T cells in the TILs-initial samples **(D)** but no correlation was observed with the percentage of CD8+ T cells **(E)**. TILs samples derived from BC biopsies and control samples used are summarized in **Supplementary Tables 1** and **2**, respectively. Values from each sample are available in **Supplementary Data**. ns (not significant), p >0.05; *, p ≤ 0.05; **, p ≤ 0.01.

The mean diversity in the TILs-initial samples was 0.45 ± 0.2 for the TRB CDR3nt sequences and did not significantly differ from TILs-CD4+ and TILs-CD8+ samples, with a mean diversity of 0.38 ± 0.22 and 0.51 ± 0.2, respectively (**Figure 3A**). Suspecting that the broad dispersion in the TCR diversity values may mask real diversity differences, we stratified data by biopsy-slices and compared diversity indexes in both subsets. Using this approach, TILs-CD8+ samples showed a significantly wider repertoire diversity than TILs-CD4+ samples (p=0.02) (**Figure 3B**). Similar results were obtained when TRA CDR3 sequences were studied (**Supplementary Figures 4A, B**).

To check whether the low TCR diversity observed in CD4+ TILs was already present in the initial cultures and facing the limitation of not having sorted CD4+ and CD8+ cells, we used the proportion of both subsets in the initial cultures to infer whether the different degrees of diversity were correlated with their presence. Diversity kept negative and significant correlated with the CD4 proportion (and positive but not significant with CD8), indicating that differences in diversity were already present in the initial cultures before their expansions (**Figures 3D, E**). Therefore, we concluded that CD4+ TILs present less diverse repertoires, and this feature can be observed in the initial cultures when they are mainly composed of CD4+ T cells.

In parallel, TCR diversity was analyzed in control samples (**Figure 3C**). No significant differences were found in PBMCs nor in the e-PBMCs samples. Moreover, the expansion method used did not significatively bias the diversity in PBMCs samples (**Supplementary Figure 5A**). We finally compared the TCR diversity in expanded T cells from PBMCs and TILs, and the TCR diversity was significantly different (**Supplementary Figure 5C**) as expected, since the peripheral diversity in healthy donors should be much broader than that in any infiltrate.

### 3.3 The lower diversity found in CD4+ TILs could not be attributed to the expansion of public sequences or to higher convergence

**Figure 4:**
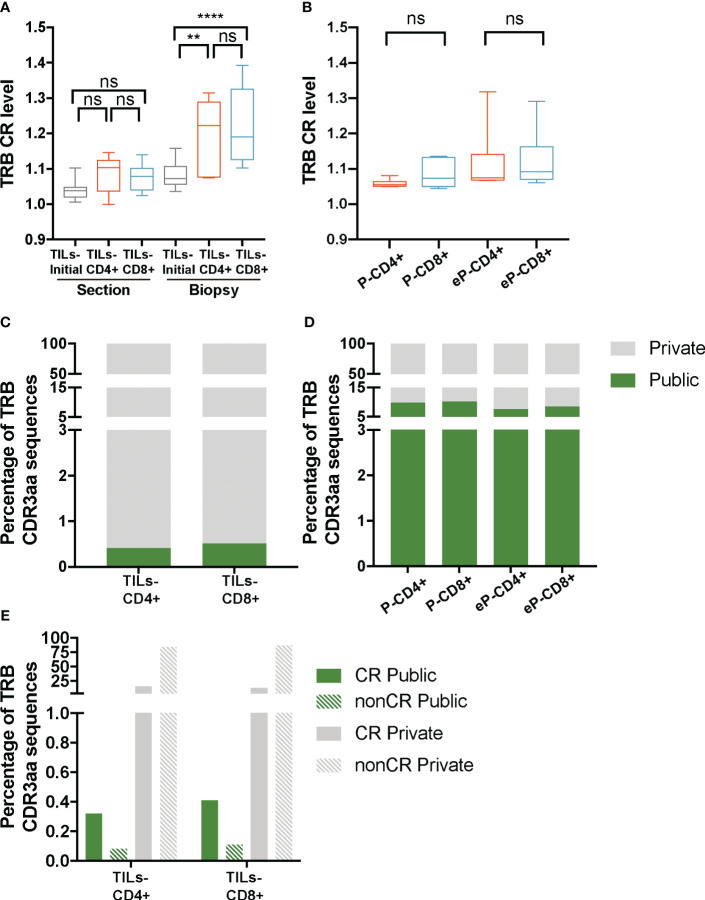
TRB CR level and percentage of public sequences in TILs and peripheral T cells from healthy donors. **(A)** The TRB CR level of TILs samples calculated by biopsy and by section (slices) did not show significant differences between different groups of samples. In the biopsy analysis, a significantly higher CR level was observed bot in the TILs-CD4+ and the TILs-CD8+ samples compared to the TILs-initial samples, indicating that certain convergent sequences in different slices were selected after expansions. **(B)** No differences were observed in the CR level of T cells from PBMCs, neither before (P-CD4+ vs. P-CD8+) not after (eP-CD4+ vs. eP-CD8+) *in vitro* expansions. **(C)** Similar percentages of TRB public sequences were observed in TILs-CD4+ and TILs-CD8+ samples. **(D)** The percentage of TRB public sequences in PBMCs was higher than in TILs, but no differences were observed between CD4+ and CD8+ T cells, neither before (P-CD4+ vs. P-CD8+) not after (eP-CD4+ vs. eP-CD8+) *in vitro* expansions. **(E)** The percentage of TRB public sequences was higher among the convergent compared to the non-convergent group of sequences in both TILs groups of samples. TILs samples derived from BC biopsies and control samples used are summarized in **Supplementary Tables 1** and **2**, respectively. Values from each sample are available in **Supplementary Data**. CR, convergent sequences; nonCR, nonconvergent sequences. ns (not significant), p >0.05; **, p ≤ 0.01; ***, ****, p ≤ 0.0001.

Lower diversity or higher clonality may result from the selection of certain clonotypes in the tumor infiltrates. Therefore, the lower diversity observed in the TILs-CD4+ samples could indicate a more restricted selection in this subset, which could be a result of a higher degree of convergent recombination (CR), i.e., several CDR3nt encoding the same CDR3aa sequences due to TCR selection during the immune response. As for diversity, for the CR analysis data were stratified both by biopsy-slices and by biopsy. The average TRB CR levels obtained from the TILs-initial samples were 1.05 ± 0.02 in the biopsy-slice analysis and 1.1 ± 0.04 in the biopsy analysis. In this latter, the convergence of TRB sequences increased significantly in both TILs-CD4+ and TILs-CD8+ when compared with TILs-initial samples (p=0.006 and p<0.0001, respectively) (**Figure 4A**), suggesting that *in vitro* expansions may affect the convergence level by selecting some given clones in different biopsy-slice cultures. In the control analysis, we did not find any difference in the CR level between different samples (**Figure 4B**). Moreover, the expansion method did not affect the CR level and similar results were obtained in eP-CD4+ and eP-CD8+ and TILs-CD4+ and TILs-CD8+ samples (**Supplementary Figures 5B, D**, respectively).

As the CR level could not explain the lower diversity observed in TILs-CD4+ samples, we investigated whether the restricted repertoire was caused by a higher proportion of TRB CDR3aa shared sequences among two or more biopsies (**Figure 4C**); from now on, referred to as public sequences for those shared among at least two biopsies/patients and to as private sequences for those only found on one biopsy. The number of public sequences in TILs-CD4+ and TILs-CD8+ samples were 17 and 12, respectively, representing only 0.4% of the total TRB CDR3aa sequences in both cases. Sequences were shared between a maximum of three biopsies, although the majority were found in only two biopsies. There was no association of biopsy histological type with shared public sequences (**Supplementary Figure 6**). A higher percentage of public sequences was obtained in the control samples (7-10%, **Figure 4D**), probably due to the higher number of sequences obtained.

As the difference in the number public sequences within each subset was not relevant and there was not an obvious association with the diversity, we compared we compared normalized fractions of the different public sequences found in the biopsies. Notably, most of the public sequences, were present at low frequencies (<0.005%) (**Supplementary Figures 7A-D**) but similarly observed in both TILs-CD4+ and TILs-CD8+ samples. Interestingly, most of the private sequences were non-convergent i.e., codified by a unique CDR3nt sequence, whereas most of the public sequences were convergent (**Figure 4E**). Therefore, we concluded that the CR level was associated to the proportion of public sequences, as these were usually more convergent, but none of these features were associated with TCR diversity.

### 3.4 Expanded CD4+ T-TILs present similar TCRs with conserved CDR3aa motifs shared among studied patients

**Figure 5:**
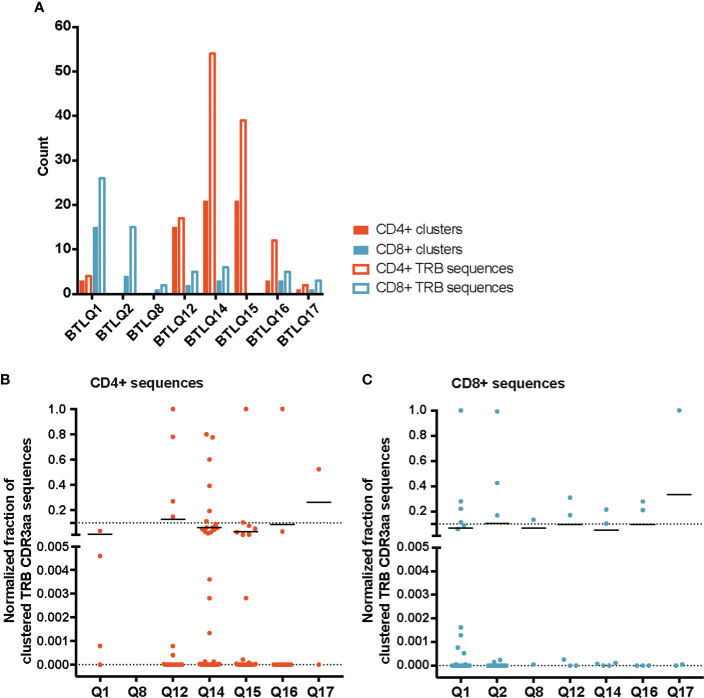
TRB CDR3aa motifs in TILs-CD4+ and TILs-CD8 samples. **(A)** Number of motifs obtained by GLIPH2 (16, 17) and the number of TRB CDR3 sequences comprising them in the different biopsies analyzed. Normalized fractions of clustered TRB sequences in the TILs-CD4+ **(B)** and the TILs-CD8+ **(C)** samples. A higher number of motifs composed by a higher number of sequences was obtained among TILs-CD4+ compared to TILs-CD8+ samples. Only those motifs with a Fisher’s exact test score and expansion score <0.5 were selected. Note that the absence of CD4+ and CD8+ clusters in the BTLQ2 and BTLQ15 biopsies, respectively, was due to the lack of samples in these biopsies as purified populations could not be obtained.

**Figure 6:**
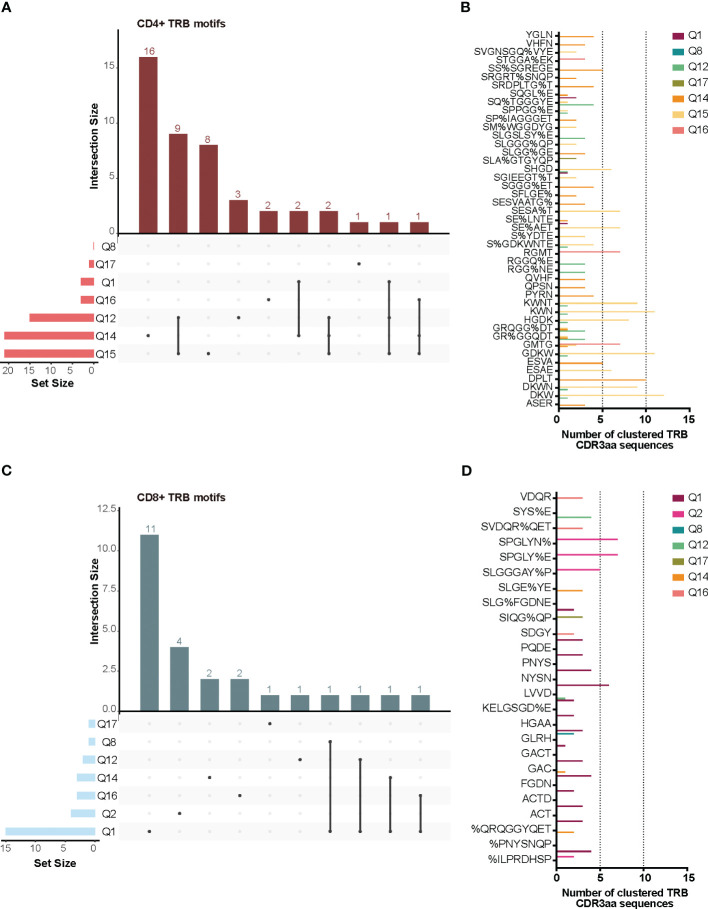
TRB CDR3aa shared motifs and amino acid sequences. UpsetPlots show the number of motifs found in the TILs-CD4+ **(A)** and the TILs-CD8+ **(C)** in each biopsy and their exclusivity and/or privacy in the different biopsies analyzed. A higher number of shared motifs was observed among TILs-CD4+ samples. Amino acid sequences of motifs found in the TILs-CD4+ **(B)** and TILs-CD8+ **(D)** samples and the number of CDR3aa sequences containing each motif. GLIPH2 (16, 17) was used for clustering and only those motifs with a Fisher’s exact test score and expansion score <0.5 were selected.

TCR similarity may be due to common 3-5-mer motifs in the CDR3aa sequence. The GLIPH2 (16, 17) algorithm identifies clusters of TRB CDR3aa sequences with a high probability of sharing antigen specificity by clustering short amino acid motifs on the CDR3. We applied GLIPH2 (16, 17) considering only significant motifs (Fisher’s exact test) with an expansion score >0.5. The number of motifs identified among TILs-CD4+ was clearly larger than in TILs-CD8+ sequences (45 *vs.* 25) (**Figure 5A**). Several motifs were present in a high fraction of TCRs and similarly distributed after normalizing by the sample size in both sample subtypes (**Figures 5B**, **C**). Of the 45 motifs detected among CDR3aa sequences of TILs-CD4+ samples, 15 were present in at least two different biopsies (**Figure 6A**). Several motifs were present in five CDR3aa sequences and some of them even in more than ten (**Figure 6B**). In TILs-CD8+ samples, only four motifs were found in more than one biopsy (**Figure 6C**), and the most frequent motif was found in seven CDR3aa sequences (**Figure 6D**). We ruled out the possibility that the sample size (number of sequences) was responsible for the larger number of motifs in TILs-CD4+ samples by analyzing the correlation between the number of reads and sequences with the number of total and shared motifs (**Supplementary Figure 8**). We therefore concluded that CD4+ TILs were not only less diverse but also more similar intra- and inter-individually. Finally, although no relationship was found between motifs and the histological type of breast tumors, it is noteworthy that the two biopsies with a greater number of motifs among TILs-CD8+ samples corresponded to TN cases.

### 3.5 TCR motifs may be used to identify tumor-reactive CD4+ T cells

**Figure 7:**
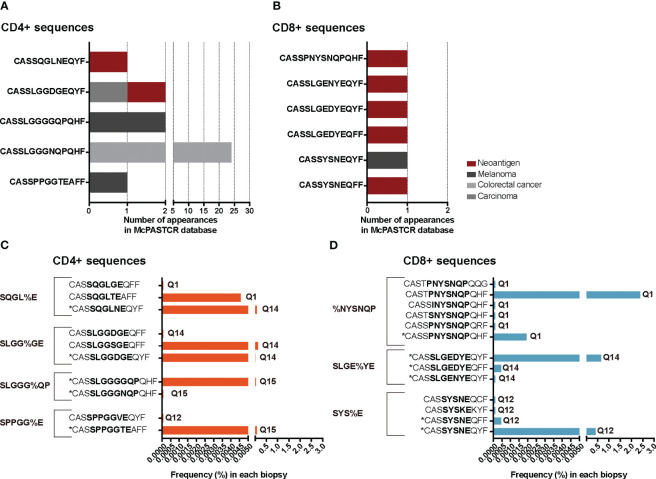
TRB CDR3 sequences found in the McPAS-TCR (18) database and annotated sequences using motifs. The number of appearances of sequences described in the literature as tumor-specific, grouped by the cancer type or antigen etiology in TILs-CD4+ **(A)** and TILs-CD8+ samples **(B)**. Frequency of tumor-specific annotated sequences in the TILs-CD4+ **(C)** and TILs-CD8+ samples **(D)**. Sequences with an * are those which were described in the literature, while the others are manually annotated. The sequence in bold highlights the motif contained in that cluster and the biopsies in which they were found are noted next to the frequency bars.

We reasoned that the relative homogeneity observed within each individual but also between patients in TILs-CD4+ samples may be associated to cancer-related expansions. In searching for clues supporting this possibility, three sets of sequences from our TCR CDR3aa data i.e., sequences with a frequency >0.1%, public sequences, and sequences clustered by GLIPH (16, 17) motifs, were compared with sequences reported in McPAS-TCR (18), a comprehensive database of TCR sequences of known specificity/pathological context. Those sequences from our sets which had a match in McPAS-TCR (18) were then grouped into four categories according to the context in which each sequence had been reported: allergy, autoimmunity, cancer, and pathogens. We then reclassified manually our CDR3aa sequences and those containing a motif previously identified in a cancer patient were considered putatively as tumor associated (named annotated sequences). Five motif-selected sequences corresponded to TILs-CD4+ and six to TILs-CD8+ TCRs (**Table 1**). Using TILs-CD4+ sequences, the number of matches in McPAS-TCR (18) was larger in the clustered motif-selected set than in the high-frequency or public sequence sets, but for TILs-CD8+ sequences, the larger number of matches was obtained in the frequency >0.1% set. By manual annotation, the number of sequences associated with the cancer motifs increased to 10 and 13 in the TILs-CD4+ and TILs-CD8+ samples, respectively (**Table 1**), none of them previously reported in breast cancer (**Figures 7A, B**). However, two and five sequences (from TILs-CD4+ and TILs-CD8+, respectively) have been reported as neoantigen-specific (**Figures 7A, B**). Analyzing the individual sequences associated to each motif, we found only one sequence from the TILs-CD4+ and from the TILs-CD8+ samples that qualified as high frequency but they did not correspond to sequences reported in the literature, i.e., the CASSLGGSGEQFF carrying the SLGG%GE motif found among TILs-CD4+ in the Q14 biopsy (**Figure 7C**), and the CASTPNYSNQPQHF carrying the %NYSNQP motif that found among TILs-CD8+ cells in the Q1 biopsy (**Figure 7D**). Among TILs-CD4+, two of the four motifs were associated to sequences found in two different biopsies, but in the TILs-CD8+ group, each cluster of sequences was associated to a single biopsy (**Figures 7C, D**). Even if we only found a few matches with reported sequences, clustering by motifs lead to identify more clonotypes than using the sets of public sequences and, in the case of TILs-CD4+ samples, the motif clustering led to more matches than using the frequency >0.1% set. Therefore, motif analyses offer an advantage as a selection method by increasing the number of candidate sequences and may be especially useful for identifying CD4+ clonotypes.

**Table 1:** TRB CDR3aa sequences used in the McPAS-TCR database search and results obtained in each sample subgroup.

			Number of sequences reported in response to different pathologies^b^				Total
Sample group and total number of TRB CDR3aa sequences	Subgroups	Number of TRB CDR3aa sequences in the subgroup^a^	Allergy	Autoimmunity	Cancer	Pathogens	
CD4+ (8313)	>0.1%	94	3	6	3	16	28
	Public sequences	17	0	1	2	5	8
	Clustered	126	1	6	5	12	24
	Clustered + manual annotation	126	2	12	10	21	45
CD8+ (4863)	>0.1%	153	1	12	8	35	56
	Public sequences	12	1	4	5	7	17
	Clustered	62	2	2	6	9	19
	Clustered + manual annotation	62	6	12	13	13	44

a Number of sequences introduced in the database from three different subgroups: sequences with a frequency >0.1%, public sequences and sequences clustered based on motifs.

b Number of total sequences found with a Levenshtein distance of 1 based on the type of response for which they have been reported: allergy, autoimmunity, cancer or in response to pathogens.

Sequences containing motifs described in the database were used to annotate as putatively specific all the rest of the sequences containing the same motif (annotated, grey cells).

## 4 Discussion

The study of the TCR has gained considerable interest in cancer research in recent years. On the one hand, TCR is the molecule conferring T cell specificity, so TCR analysis of TILs may be used to identify tumor-specific clonotypes. On the other hand, after T cell activation through pMHC complex recognition, lymphocytes undergo clonal expansion; therefore, all T cells will present the same TCR, which can be monitored through TCR sequencing. TCR repertoire analyses are mostly focused on its prognostic and/or predictive value, both for the evolution of cancer and in response to treatment; however, little is known about the differences between repertoires of distinct T cell subpopulations. As the TCR is primarily linked to the pMHC complexes, and CD4+ and CD8+ T cells recognize different MHC molecules which, in turn, present different peptides, it would be expected that the main differences in the TCR would be present when comparing these two cell subtypes. Moreover, CD4+ and CD8+ T cells are transversal T cell subsets expected to be present in all biopsies, unlike other cellular subsets expressing certain markers which are not exclusive and may not be present in all biopsies. Therefore, we compared *in vitro* expanded CD4+ and CD8+ TILs from different BC subtypes to identify differences in their TCR repertoires.

One of the main issues in obtaining TILs is the low quantity of cells that can be extracted from a biopsy, which limits the possibility of performing several phenotypic and functional studies in parallel. Thus, T cells are usually expanded to obtain a higher quantity. In our case, a limited number of T cells did not allow for separation through a sorter to subsequently obtain a library of their TCRs. However, during the expansions, the original repertoire could be biased due to a greater proliferative capacity of certain clones, which has been recently demonstrated by Poschke et al. (24). The same authors also observed that the expansion of TILs from different sections of the same tumor results in highly divergent repertoires. Therefore, to overcome this limitation, small sections of the same biopsy were cultured allowing for several TIL samples from the same tumor, but different in their TCR repertoire and covering a larger part of the original sample. In order to determine if *in vitro* expansions modified the repertoire, biasing the results, expanded samples were compared to those of the TILs-initial samples and to control samples (peripheral T cells from healthy donors). It should be noted that the method used for the expansions was not a rapid expansion protocol (REM) nor high doses of IL-2 were used. Finally, data were both analyzed together and stratified by the BC subtype (data not shown), but we did not find any significant difference related with the latest, therefore all data were merged.

We analyzed several CDR3 features, from which we could draw some conclusions. First, shorter TRB NDNnt sequences were observed in TIL-CD8+ samples, but not in the control analysis using peripheral T cells from healthy donors, neither before nor after expansion. Some studies have examined the CDR3 length indicating that it is a dynamic property influenced by the interaction of the TCR with pMHC complexes. Particularly, it has been reported that the CDR3 undergoes a sequence-shortening process during thymic development potentially associated with MHC restriction (25). While this process is supposed to occur in all thymocytes, some studies have shown differential shortening between the two lineages (26), suggesting that its length is associated with differences in TCR-peptide interactions in the binding grooves of MHC class I and class II molecules. Additionally, Hou et al. (27) observed that naïve T cells possess larger TRB CDR3 sequences compared to memory T cells. As naïve T cells represent the entire potential repertoire, while memory cells represent the subset that has encountered specific antigens, the selection of clonotypes with a reduced CDR3 length among the latter, could imply a potential relationship between shorter CDR3 length and antigen affinity. Although a direct association with antigen recognition cannot be established, a clear distinction in the CDR3 length between CD8+ and CD4+ TILs was observed, while it was not present in the peripheral compartment of healthy individuals. This altogether may indicate that structural variations in CDR3 length in different TIL subsets could be related to variations in the affinity for different pMHC complexes and their availability in the tumor microenvironment.

Regarding the biochemical CDR3aa properties, we only found a significant difference in the charge; specifically, sequences of CD8+ TILs and control samples presented a higher frequency of amino acids with negatively charged side chains. This property has been previously described in peripheral T cells by another group (28). Therefore, it was concluded that the TRB charge difference was intrinsic to the CD4+ and CD8+ subpopulations, although the reason for this difference in amino acid usage is unknown. Previous studies have established that TRAV and TRBV gene frequencies are shaped depending on MHC haplotypes (29), indicating the involvement of MHC molecules during TCR selection. However, it is not clear how differential amino acid usage may be involved in TCR specificity, and current knowledge about TCR with pMHC complex interactions is based on a few models. The study of biochemical properties and amino acid usage has recently been considered to improve the binding specificity predictions (30). It is known that amino acid usage is not random, so properties conferred by certain amino acids may be necessary for good recognition. For example, glycine usage is higher in the TRB CDR3 sequences, with an increasing use depending on the sequence length, and its usage confers flexibility to the loops formed in the CDR3, which may be related to being more prone to poly-specificity sequences (31–33). More recently, it was reported that hydrophobic amino acids are enriched in TRB CDR3 from Tregs, suggesting that this promotes enhanced recognition of self-peptides, leading to Treg fate during thymic selection (34). In the same study, the authors developed a scoring method based on TCR properties to test the level of Tregs in a sample (34), evidencing that a deeper understanding of the association between amino acid usage and the properties they confer on the TCR could provide information on affinity.

To date, few studies have analyzed the diversity of BC and have presented opposing results. Some authors have reported that intratumoral diversity is greater than in healthy tissues (35), whereas others have observed less diverse repertoires (36). Regarding CD4+ and CD8+ diversity, some author have reported a greater CD4+ diversity in the periphery (27, 28) and similar results have been described in lung adenocarcinoma (37), but to our knowledge, this has not been evaluated in BC. In our TCR repertoire analysis, we observed greater diversity in CD8+ samples when comparing CD4+ and CD8+ TILs from the same biopsy sections using both TRA and TRB sequences. Through the control samples analysis, we ruled out that the expansion method skewed the TCR diversity and, additionally, a higher presence of CD4+ cells correlated with lower diversity levels in the initial cultures. The diverse CD8+ TIL repertoire may be explained by a less proliferative nature of CD8+ T cells compared to that of CD4+ T cells but no differences were found in the diversity of peripheral T cells before and after *in vitro* expansion, or between CD4+ and CD8+ peripheral T cells. Therefore, the higher clonality observed in the CD4+ TILs yet from the beginning of unexpanded TILs cultures may be related with a specific TCR recognition, which was investigated in greater depth.

We determined whether the expanded clones had convergent or public sequences. No significant differences were found in the level of convergence between CD4+ and CD8+ cells in either TILs or peripheral T cells. In the biopsy analysis, the CR level was much higher on TILs-CD4+ and TILs-CD8+ samples. As it was not detected in the slice analyses or between peripheral T cells, it can be concluded that it was a result of expansion of certain convergent clones that were spatially distributed in the biopsies. Second, public sequences were not more abundant among CD4+ than CD8+ TILs, and most were not found at high frequencies. Previous studies have shown that public sequences in BC present greater convergence (11), which was also observed in our samples. In summary, the low CD4+ diversity did not seem to be related to greater convergence or expansion of public sequences.

Although the disparity in CD4+ and CD8+ TILs diversity could not be associated to other repertoire properties, it still signified a variation in the extent of oligoclonal expansions. Consequently, we aimed to investigate whether there were other potential factors that could be somehow linked to antigen recognition. In various types of pathologies, such as autoimmunity (38) or infection (16, 39), it has been reported the presence of 3-5-mer motifs in CDR3aa sequences that bind the same MHC-restricted peptide antigens. Based on this, we focused on the presence of motifs using GLIPH2 (16, 17) to identify similar CDR3 sequences in different subsets. We observed a higher number of motifs within TILs-CD4+ samples, composed by more sequences and more shared between biopsies. Diversity calculations do not take motif presence into account, as it is determined based on sequence richness and abundance within a sample. However, a higher inter- and intra-similarity among TILs-CD4+ samples would correspond to the lower diversity observed, as both suggest an increased presence of expansions that may be attributed to antigen recognition. Further supporting this hypothesis, it is worth mentioning that among TILs-CD8+ samples, a greater number of motifs was observed in TN biopsies. TN tumors are known to be more immunogenic as they exhibit a higher tumor mutational burden (40–42), which can be directly associated with the presence of tumor antigen-specific T lymphocytes. The higher presence of motifs in TILs-CD4+ samples may be useful for selecting potential tumor-reactive clonotypes if, as described in other pathologies (38) (16, 39), slightly different CDR3 sequences with identical 3-4aa motifs can recognize the same pMHC. Based on this, we used sequences grouped into clusters (containing motifs) and investigated whether they had been previously described in the literature, using the McPAS-TCR (18) database. The results obtained were biased by the number of sequences introduced, and highly unbalanced by a greater number of published studies focused on CD8+ T cell investigations. However, motifs were found to be useful for manual annotation of putative tumor-specific sequences, especially among CD4+ TILs.

The great variability of the TCR repertoire, as well as the complexity of its interactions with pMHC complexes, makes it difficult to study. However, differences found between CD4+ and CD8+ TILs could be conditioned by intrinsic recognition characteristics within these subsets. A greater TCR similarity may be associated with a higher promiscuity, indicating that several similar TCRs can recognize slightly different pMHCs. The TCR promiscuity is associated with a greater tendency for cross-reactivity, which is necessary to ensure an immune response against the wide variety of pathogens and the even larger possible derived peptides. This great variety theoretically exceeds the TCR repertoire present in an individual (43), thus requiring the existence of cross-reactive TCRs capable of recognizing the entire spectrum. Given the higher functional diversity of CD4+ cells, it could be assumed that this higher promiscuity is a characteristic of this cellular subtype and is reflected in the tumor context through the observation of a greater number of motifs. However, the cross-reactivity carries the risk of generating autoimmune events, not only directed by a specific peptide but also by the possible recognition of several peptides, as it has been described in an NOD model of diabetes (44) or in human multiple sclerosis (45, 46). By contrast, a higher promiscuity would increase the likelihood of finding tumor-specific TCRs.

Lastly, it is particularly important to consider the differential contribution of class I and class II ligandomes. Ignoring the fact that tumor cells may downregulate MHC-I expression, class I tumor antigens originate from proteins within the tumor cell. If protein abundance is not a limiting factor and it contains binding motifs for MHC-I molecules, its presentation is feasible. On the other hand, MHC-II presentation is limited, first, by the presence of APCs in the tumor microenvironment; second, by the accessibility of the antigen source protein, which is theoretically exogenous and, consequently, must be captured, processed, and presented; and third, by the anchoring motif of the MHC-II molecule. Considering these differences in presentation, a neoantigen will likely be more efficiently presented by MHC-I molecules. Conversely, antigens more likely to be presented in the context of class II would be overexpressed antigens. CD8+ T cells probably recognize neoantigens with higher affinity than self-peptides because thymic selection has supposedly eliminated clones that recognize self-pMHC with high affinity. However, it has been shown in mice that low-affinity CD8+ T cells are capable of generating an anti-tumor response only in the presence of CD4+ T cells (47).

Considering all this, in tumors with a high mutational burden, it is more likely that neoantigens that induce specific activation CD8+ T cells can be generated, even though chronic exposure can trigger mechanisms of peripheral tolerance such as PD-1 expression. In this case, favoring a CD8+ response, as well as the use of ICIs that can reactivate it, can help to eliminate the tumor. In tumors in which the antigens directing the immune response are tumor-associated and, therefore, self-antigens, a differential presentation associated with the dose may be necessary to break tolerance. In these cases, activation of CD4+ T cells may occur more easily due to less restrictive recognition. This scenario could also contribute to Treg activation since they are known to present TCRs with a higher affinity for self-pMHC complexes (34). Recently, Rosenberg's group used TCR gene therapy derived from a Treg clonotype to drive the response to the germline antigen MAGE-A3, and demonstrated its safety and efficacy in metastatic cancers of different origins (14). In conclusion, in this study, we found differences in the TCR repertoire between CD4+ and CD8+ T cells, which may be associated with their recognition abilities and cellular functions. From our results, we can conclude that the CD4+ T cell response is more homogeneous among individuals, whereas the CD8+ T cell response seems to be more limited to more immunogenic tumors, such as TNBC. This may be especially relevant in selecting an effective personalized treatment based on the antigen driving the immune response: class I and class II-restricted TCRs may be used to target tumor-specific and tumor-associated antigens, respectively.

## Data availability statement

The datasets presented in this study can be found in online repositories. The names of the repository/repositories and accession number(s) can be found below: https://www.ncbi.nlm.nih.gov/, PRJNA925311 https://www.ncbi.nlm.nih.gov/, PRJNA759174.

## Ethics statement

Breast cancer patient biopsies were obtained from surplus hospital material donated by Hospital Quirón of Barcelona and Hospital Vall d’Hebron using standard surgical procedures, with the appropriate approval of the Ethical and Scientific Committee of the institutions. Consent was obtained from patients according to the local institutional review board requirements.

## Author contributions

Conceptualization: AA and MM. Methodology: AA, GL, EM, and FA. Investigation: AA, GL, EM, FA, VM, and VP. Resources: VM, VP, MG, LG, and JP. Writing—original draft: AA and MM. Writing—review and editing: AA, JC, and MM. Supervision: JC and MM. All authors contributed to the manuscript and approved the submitted version.
