# Supplementary material for: Analysis of tumor infiltrating CD4+ and CD8+ CDR3 sequences reveals shared features putatively associated to the anti-tumor immune response
Source: Front Immunol. 2023 Aug 4;14:1227766. doi: 10.3389/fimmu.2023.1227766 (PMC10436466; doi:10.3389/fimmu.2023.1227766)
Supplement: Supplementary file 2 [file DataSheet_2.docx]

Supplementary Material

Analysis of tumor infiltrating CD4+ and CD8+ CDR3 sequences reveals shared features putatively associated to the anti-tumor immune response

Andrea Aran, Gonzalo Lázaro, Vicente Marco, Elisa Molina, Ferran Abancó, Vicente Peg, María Gión, Laia Garrigós, José Pérez, Javier Cortés, Mercè Martí*

*** Correspondence:** Mercè Martí: merce.marti@uab.cat

# Supplementary Figures and Tables

## Supplementary Figures

##
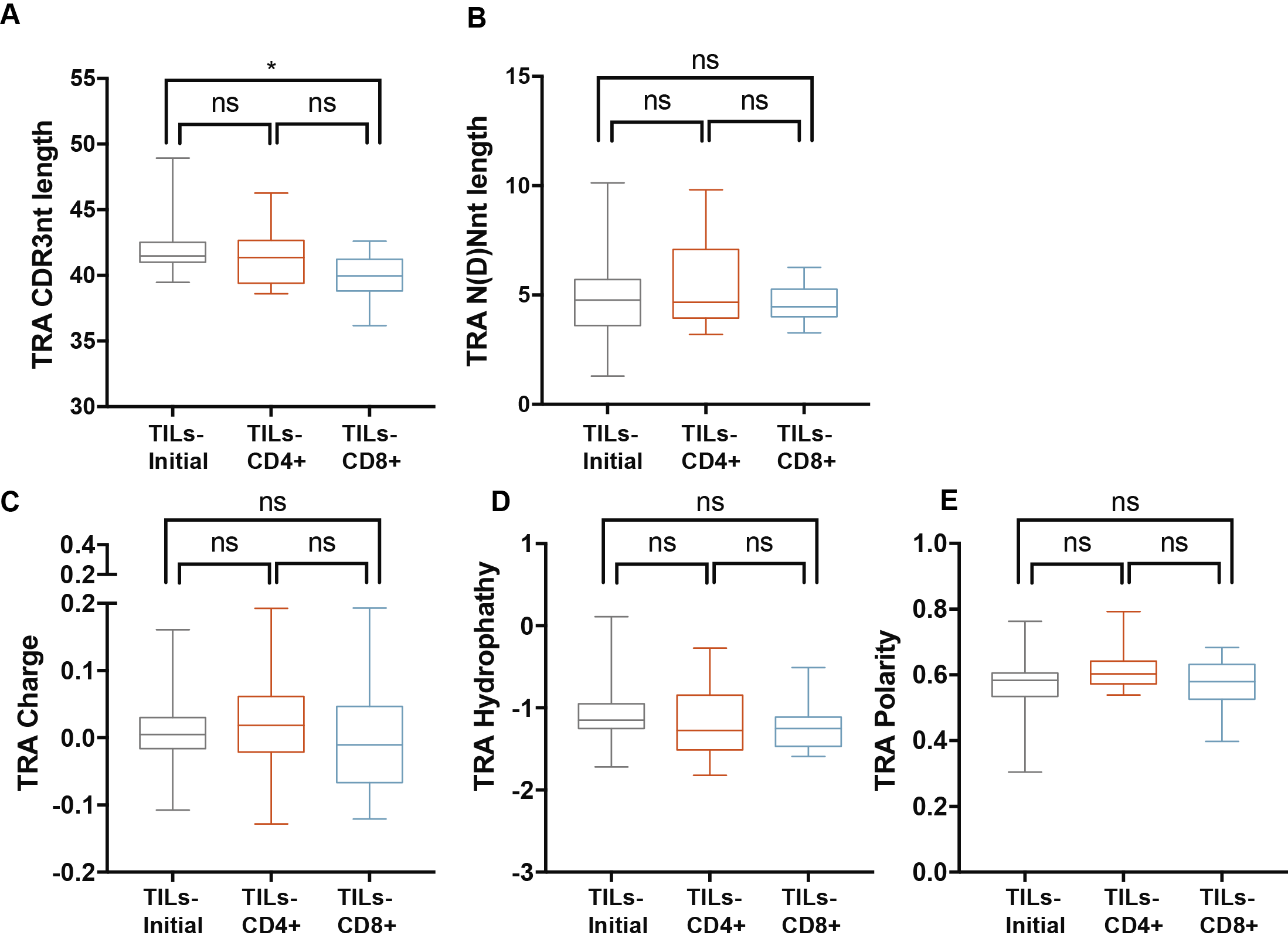


**Supplementary Figure 1. TRA physicochemical properties of TILs-initial, TILs-CD4+ and TILs-CD8+ samples.** CDR3nt length (A), NDNnt length (B) of TRA CDR3nt sequences. TILs-initial samples showed a significantly larger CDR3nt length compared with TILs-CD8+ samples. Biochemical properties (charge (C), hydropathy (D) and polarity (E)) of the central 5-mer TRA CDR3aa. Data were weighted by the frequency of sequences and normalized by the subregion size (5aa). None of the biochemical properties showed significant differences between groups. TILs samples derived from BC biopsies used are summarized in Supplementary Table 1. Values from each sample are available in Supplementary Data. ns (not significant), p >0.05; *, p ≤ 0.05.


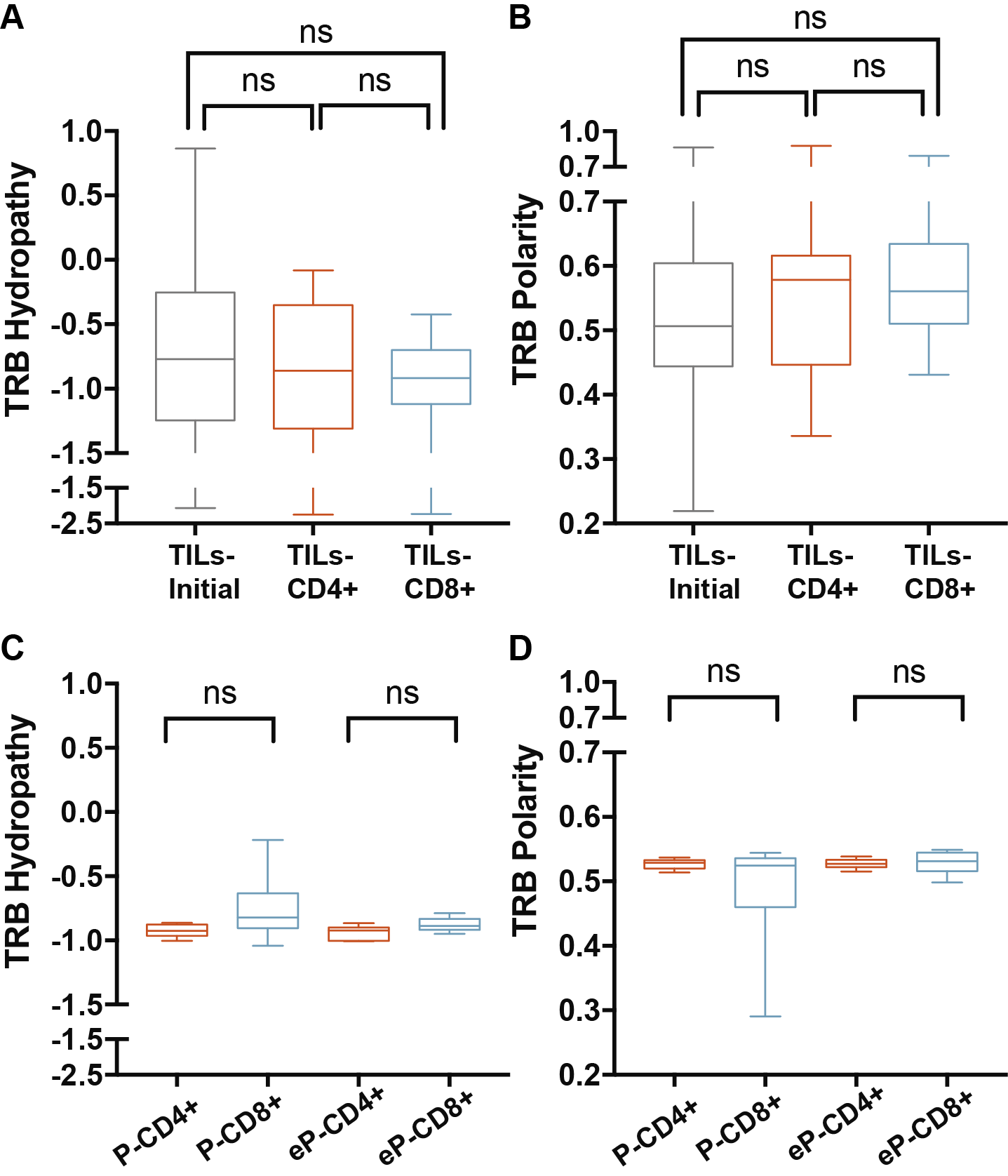


**Supplementary Figure 2. TRB biochemical properties of TILs and PBMCs samples.** Hydropathy and polarity of the central 5-mer TRB CDR3aa of TILs samples (A, B) and PBMCs from healthy donors (C, D). CD4+ and CD8+ cells derived from PBMCs were analyzed before (P-CD4+ and P-CD8+) and after (eP-CD4+ and eP-CD8+). *in vitro* expansions. Data were weighted by the frequency of sequences and normalized by the subregion size (5aa). None of the biochemical properties analyzed showed significant differences between groups neither in TILs nor in PBMCs. TILs samples derived from BC biopsies and PBMCs from healthy donors used are summarized in Supplementary Table 1 and 2, respectively. Values from each sample are available in Supplementary Data. ns (not significant), p >0.05.


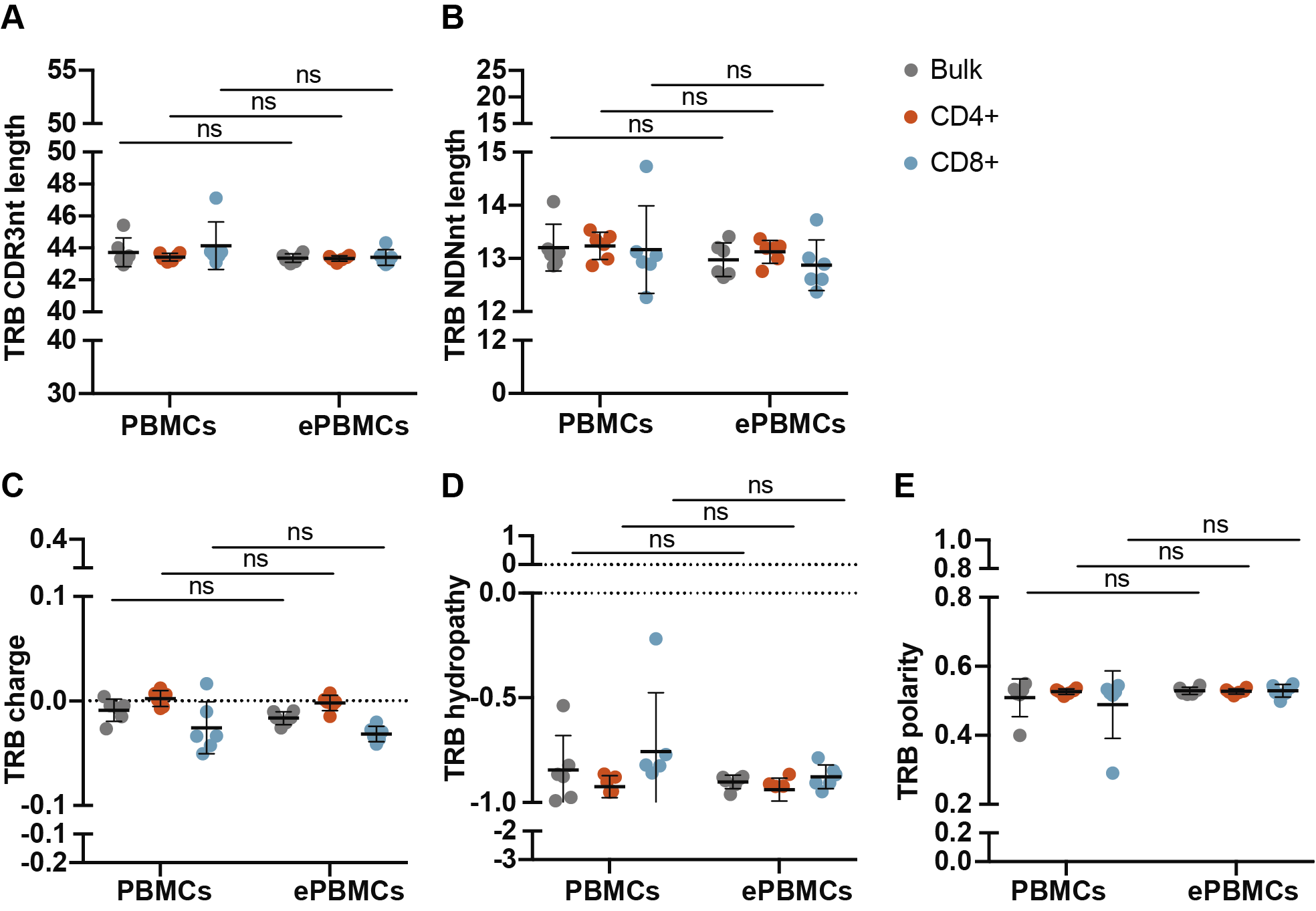


**Supplementary Figure 3. TRB physicochemical properties of peripheral T cells from healthy donors before and after *in vitro* expansions.** CDR3nt length (A), NDNnt length (B), charge (C), hydropathy (D) and polarity (E) of TRB CDR3aa sequences from all PBMCs samples before and after *in vitro* expansions. For the biochemical properties (C-E), data were weighted by the frequency of sequences and normalized by the subregion size (central 5-mer of TRB CDR3aa). None of the properties analyzed showed significant differences between groups of samples before and after expansions, indicating that the expansion method did not bias these TRB properties. PBMCs samples from healthy donors are summarized in Supplementary Table 2. Values from each sample are available in Supplementary Data. ns (not significant), p >0.05.


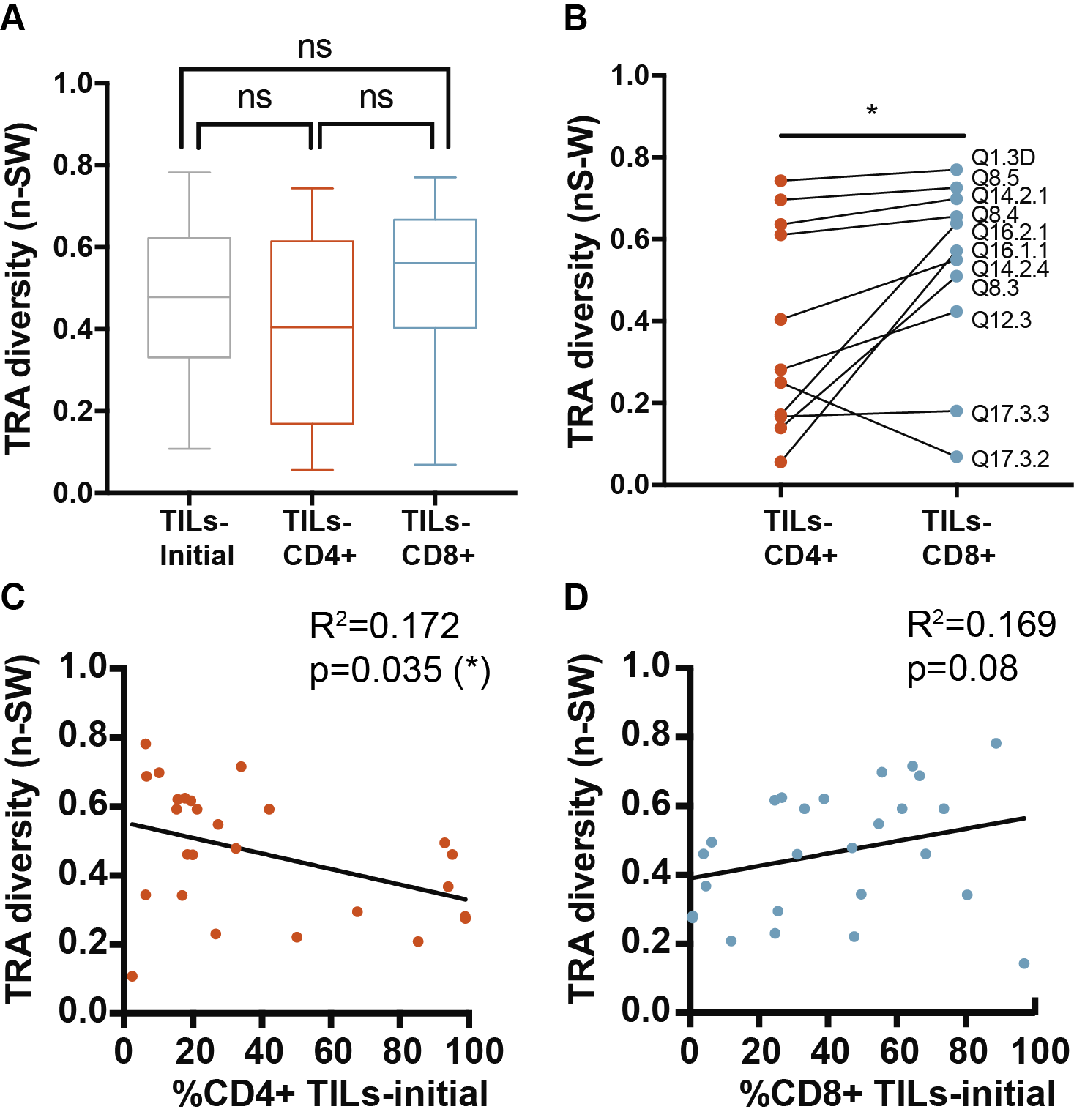


**Supplementary Figure 4. TRA diversity in TILs samples.** TRA diversity was calculated using the normalized Shannon-Wienner index (nS-W). (A) TRA diversity analysis calculated using all samples did not show differences between groups. (B) The paired analysis of TRA diversity calculated using only the values from slices from which TILs-CD4+ and TILs-CD8+ samples were available revealed a significant higher diversity in the CD8+ group. (C) A significant inverse correlation of the percentage of CD4+ T cells in the TILs-initial cultures with the TRA diversity was observed. (D) The percentage of CD8+ T cells in the TILs-initial cultures did not correlate with the TRA diversity. CD4+ and CD8+ T cell abundances were analyzed by flow cytometry and HTS was performed using cells collected on the same day. TILs samples derived from BC biopsies used are summarized in Supplementary Table 1. Values from each sample are available in Supplementary Data. ns (not significant), p >0.05; *, p ≤ 0.05.


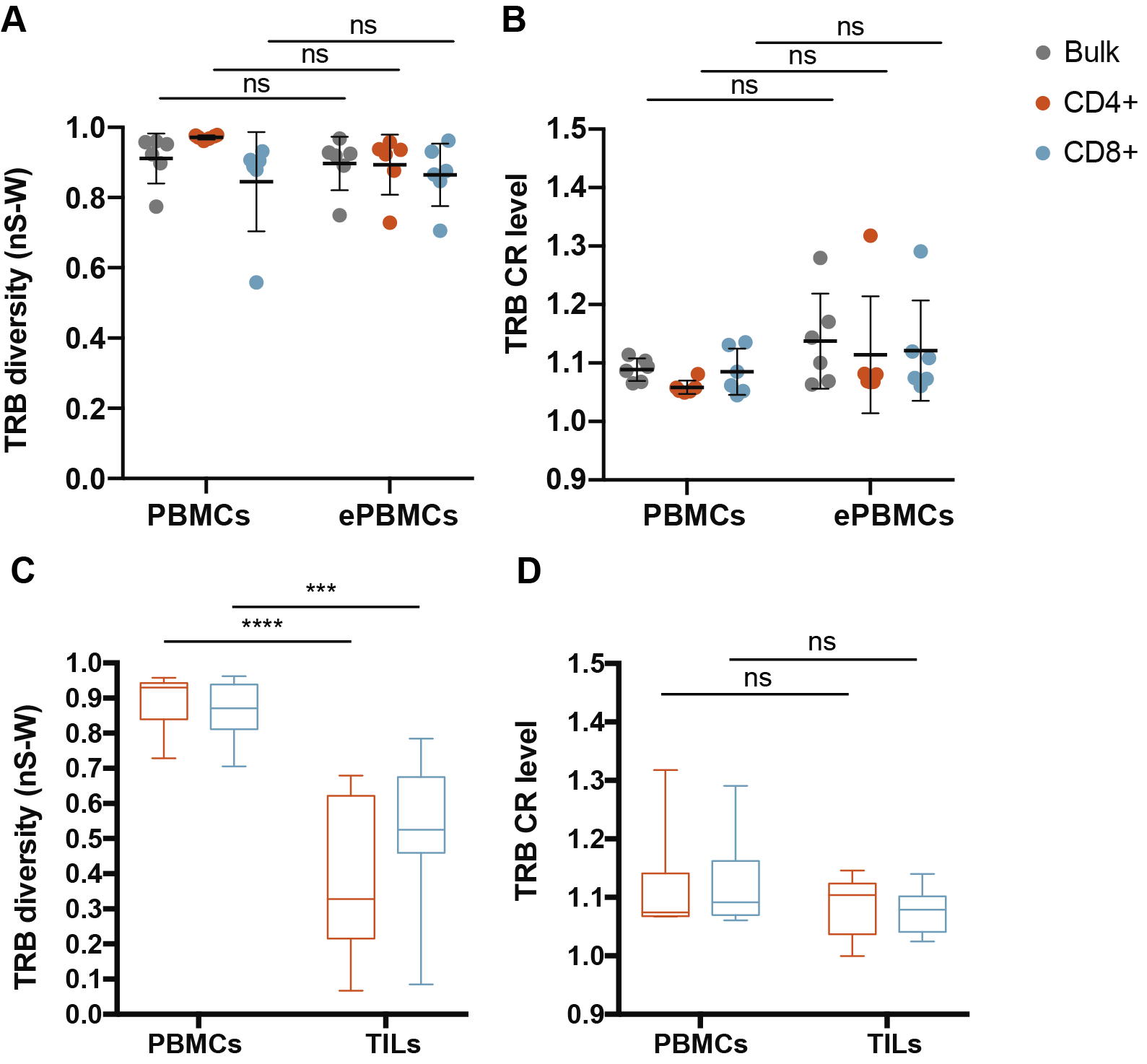


**Supplementary Figure 5. TRB repertoire properties of peripheral T cells from healthy donors before and after *in vitro* expansions.** TRB diversity (A) and convergent recombination (CR) level (B) analysis did not show significant differences before and after *in vitro* expansions, indicating that the expansion method did not bias these TRB properties. The TRB diversity level was significantly higher in PBMCs samples compared to TILs samples (C) but the CR level was similar in both groups of samples (D). TILs samples derived from BC biopsies and PBMCs from healthy donors used are summarized in Supplementary Table 1 and 2, respectively. Values from each sample are available in Supplementary Data. ns (not significant), p >0.05; *, p ≤ 0.05; **, p ≤ 0.01; ***, p ≤ 0.001; **** p ≤ 0.0001.

**
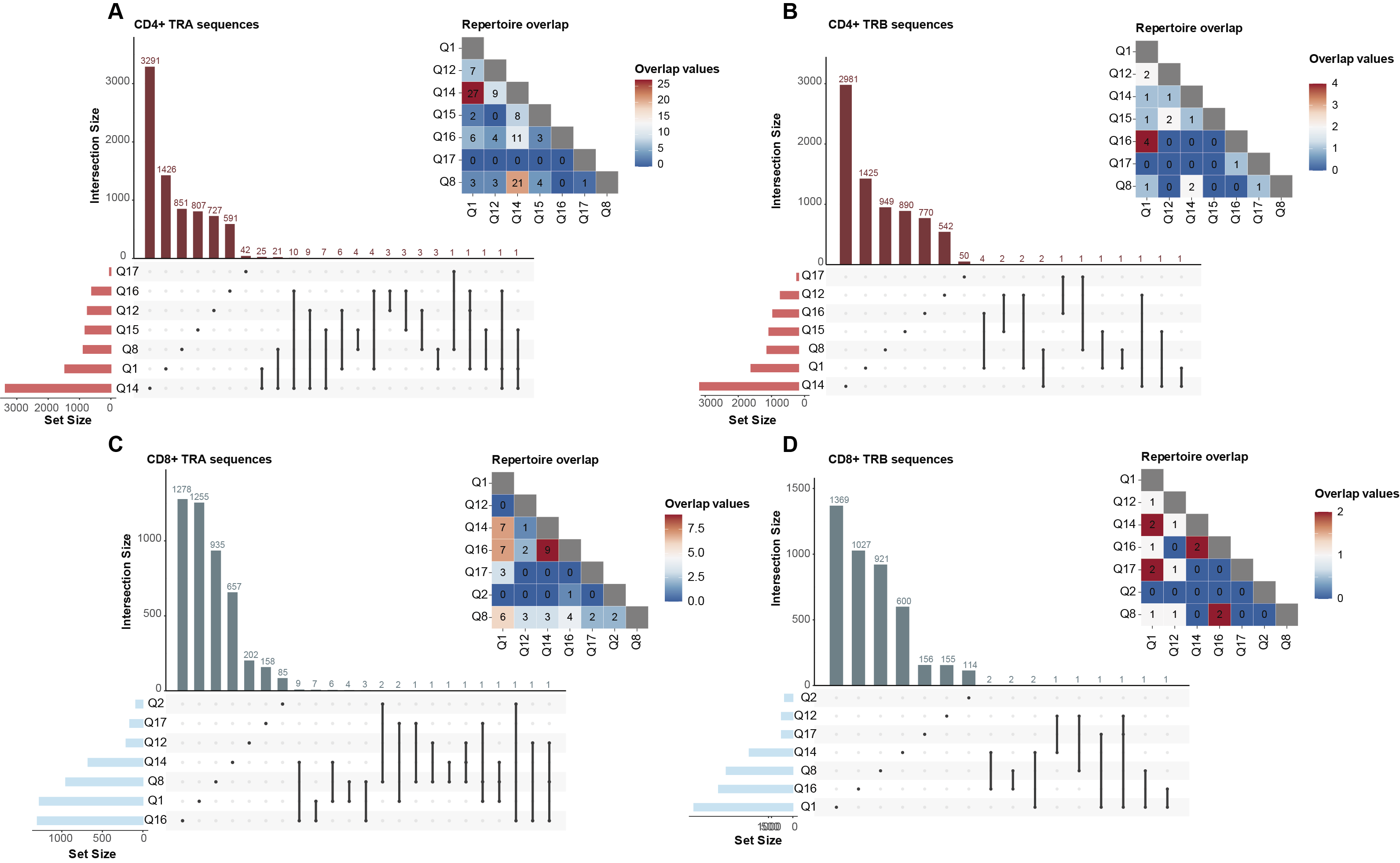
Supplementary Figure 6. TCR repertoire overlap between biopsies in the CD4+ and CD8+ groups of TILs samples.** UpsetPlots show the number of sequences identified in each biopsy in the CD4+ and CD8+ groups of samples, among TRA (A and C) and TRB (B and D) sequences, respectively. The overall repertoire overlap values are shown on the right of UpsetPlots.

**
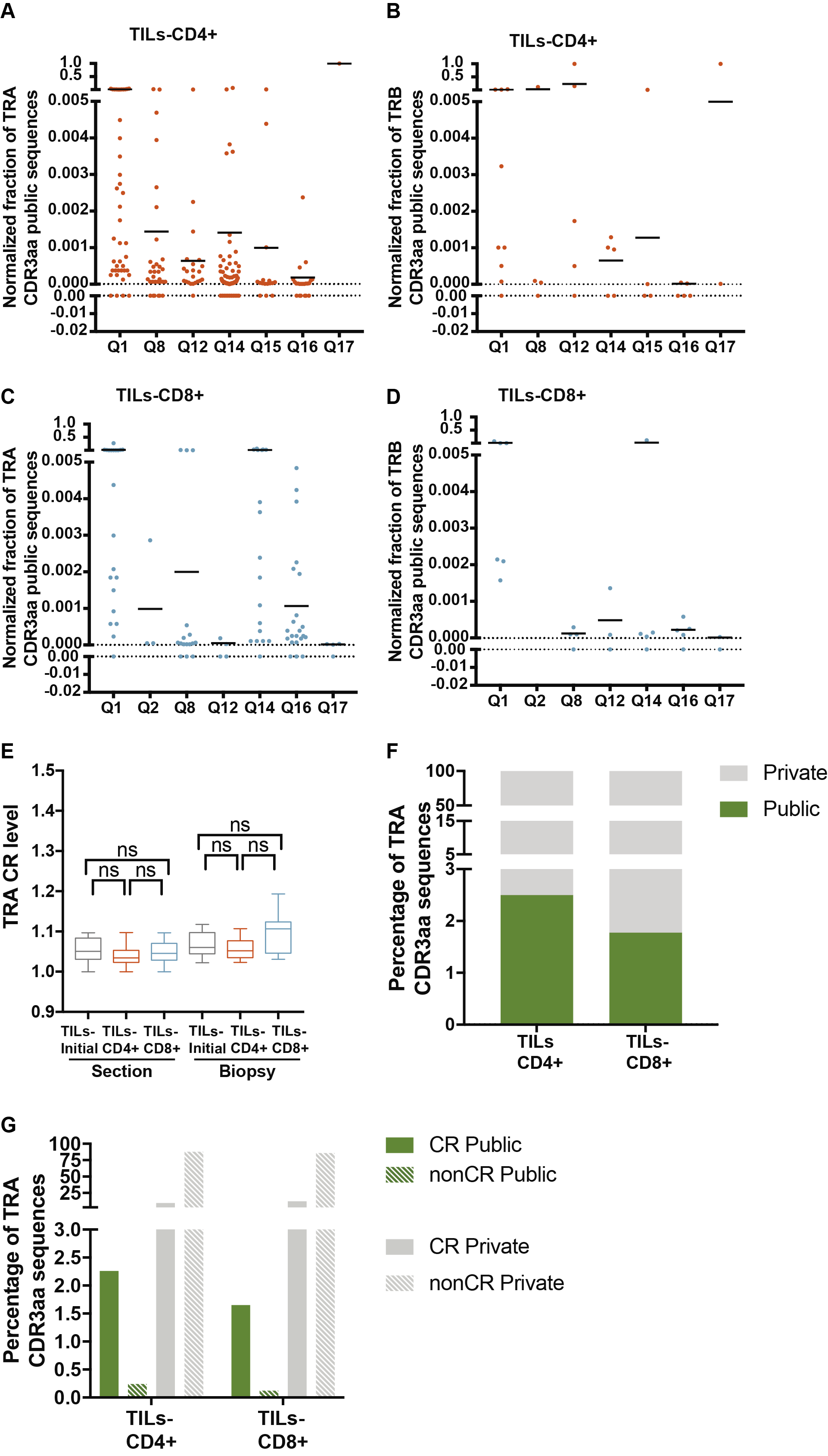
**

**Supplementary Figure 7. Public sequence description from TILs-CD4+ and TILs-CD8+ samples.** The normalized fraction of TRA and TRB public sequences in the CD4+ (A, B) and CD8+ (C, D) groups of samples revealed a similar number of high-abundant public sequences. (E) TRA CR level of TILs samples calculated by biopsy and by section (slices) did not show significant differences between different groups of samples. (F) Similar percentages of TRA public sequences were observed in TILS-CD4+ and TILs-CD8+ samples. (D) The percentage of TRA public sequences was higher among the convergent compared to the non-convergent group of sequences. CR, convergent sequences; nonCR, nonconvergent sequences. ns (not significant), p >0.05.


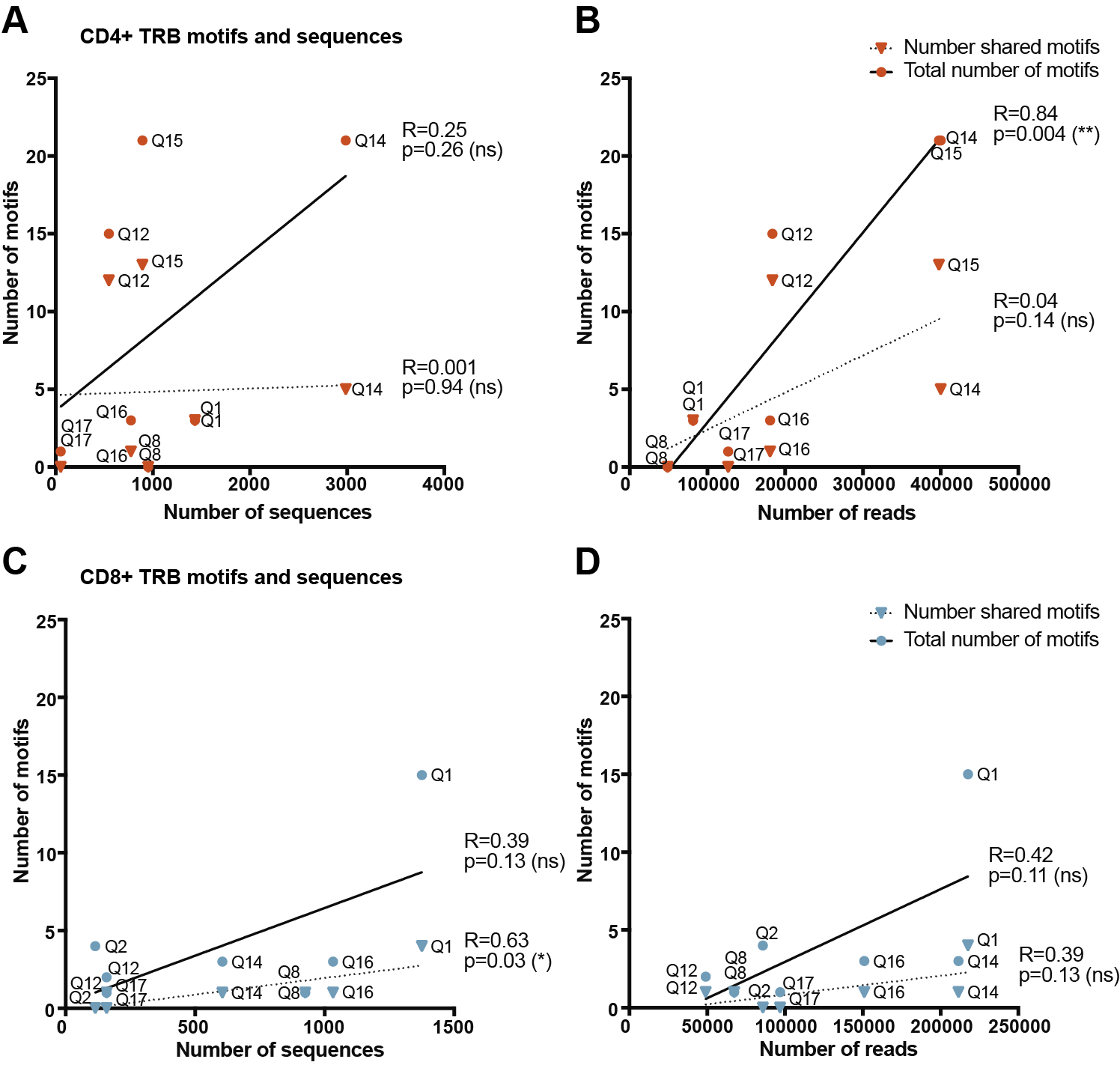


**Supplementary Figure 8. Correlation of the number of motifs identified among TRB sequences and the total number of sequences and reads.** Correlations in the CD4+ and in the CD8+ groups of samples of number of sequences (A, C) and reads (B, D) with the total number of motifs (continuous line) and the number of shared motifs between at least two different biopsies (dotted line). The number of shared motifs did not correlate with the number of sequences and reads in the TILs-CD4+ samples, although the total number of motifs showed a significant correlation with the number of reads obtained in this group. In the TILs-CD8+ samples, the number of sequences positively correlated with the number of shared motifs. TILs samples derived from BC biopsies used are summarized in Table 1. ns (not significant), p >0.05; *, p ≤ 0.05; **, p ≤ 0.01.

## Supplementary Tables

**Supplementary Table 1. Breast cancer samples used for the TCR HTS analysis and number of CDR3nt sequences obtained.**

|  |  |  |  |  | **Number of CDR3nt sequences^b^** | | | | | |
| --- | --- | --- | --- | --- | --- | --- | --- | --- | --- | --- |
| **Breast Cancer type** | **Biopsy** | **Age** | **Infiltration level of TILs^a^** | **Section of the biopsy (samples)** | **Initial TILs cultures** | | **Expanded and sorted TILs** | | | |
|  |  |  |  |  |  |  | **CD4+** | | **CD8+** | |
|  |  |  |  |  | **TRA** | **TRB** | **TRA** | **TRB** | **TRA** | **TRB** |
| TN | BC-PS-562 (562) | 44 | High | 562.4 | 3204 | 1601 | ND | ND | ND | ND |
|  |  |  |  | 562.6 | 10046 | 4317 | ND | ND | ND | ND |
|  |  |  |  | 562.7 | 1079 | 1130 | ND | ND | ND | ND |
|  |  |  |  | 562.11 | 2085 | 896 | ND | ND | ND | ND |
| TN | BTLQ1 (Q1) | 58 | Inter | Q1.1B | 390 | 439 | ND | ND | ND | ND |
|  |  |  |  | Q1.3D | 8 | 220 | 1625 | 1552 | 1296 | 1308 |
|  |  |  |  | Q1.4B | 589 | 1025 | ND | ND | 145 | 205 |
|  |  |  |  | Q1.8D | 86 | 167 | ND | ND | ND | 153 |
|  |  |  |  | Q1.9D | 287 | 314 | ND | ND | ND | ND |
| TN | BTLQ2 (Q2) | 61 | Inter | Q2.2M | 1931 | 2827 | ND | ND | ND | ND |
|  |  |  |  | Q2.8D | 1336 | 1364 | ND | ND | 105 | 149 |
| LB | BTLQ7  (Q7) | 43 | High | Q7.5 | 2666 | 3119 | ND | ND | ND | ND |
|  |  |  |  | Q7.6 | 3617 | 4083 | ND | ND | ND | ND |
|  |  |  |  | Q7.8 | 2520 | 3255 | ND | ND | ND | ND |
| LB | BTLQ8  (Q8) | 42 | Low | Q8.3 | 731 | 691 | 68 | 64 | 326 | 261 |
|  |  |  |  | Q8.4 | 1560 | 1795 | 632 | 709 | 534 | 491 |
|  |  |  |  | Q8.5 | 1028 | 1095 | 256 | 297 | 263 | 378 |
| TN | BTLQ10  (Q10) | 63 | Inter | Q10.1 | 745 | 965 | ND | ND | ND | ND |
|  |  |  |  | Q10.3 | 370 | 532 | ND | ND | ND | ND |
|  |  |  |  | Q10.5 | 1673 | 1703 | ND | ND | ND | ND |
| LB | BTLQ12  (Q12) | 55 | Inter | Q12.3 | ND | ND | 332 | 299 | 220 | 220 |
|  |  |  |  | Q12.4 | 1737 | 1491 | ND | ND | ND | ND |
|  |  |  |  | Q12.5 | ND | ND | 473 | 428 | ND | ND |
| LA | BTLQ14  (Q14) | 48 | Inter | Q14.2.1 | ND | ND | 1289 | 1474 | 538 | 532 |
|  |  |  |  | Q14.2.2 | ND | ND | 1202 | 1083 | ND | ND |
|  |  |  |  | Q14.2.3 | ND | ND | 560 | 462 | ND | ND |
|  |  |  |  | Q14.2.4 | ND | ND | 778 | 828 | 280 | 331 |
| LA | BTLQ15  (Q15) | 55 | Low | Q15.2.1 | 118 | 92 | 229 | 404 | ND | ND |
|  |  |  |  | Q15.2.2 | ND | ND | 566 | 508 | ND | ND |
|  |  |  |  | Q15.2.3 | 80 | 407 | 80 | 198 | ND | ND |
| LA | BTLQ16  (Q16) | 63 | Inter | Q16.1.1 | ND | ND | 348 | 543 | 1173 | 945 |
|  |  |  |  | Q16.2.1 | 1139 | 1125 | 320 | 316 | 384 | 376 |
| LB | BTLQ17  (Q17) | 74 | Low | Q17.3.2 | 568 | 539 | 16 | 23 | 23 | 53 |
|  |  |  |  | Q17.3.3 | ND | ND | 37 | 57 | 111 | 88 |
|  |  |  |  | Q17.3.5 | 219 | 165 | ND | ND | 62 | 79 |
| **Total** |  |  |  |  | 39812 | 35357 | 8811 | 9245 | 5460 | 5569 |

^a^TILs level of infiltration was defined according to the criteria established by Salgado et al.^50^.

^b^Number of CD3nt sequences obtained by HTS in different samples analyzed.

LA, luminal A; LB, luminal B; ND, not done; TN, triple-negative.

**Supplementary Table 2. Healthy donors’ samples used for the TCR HTS analysis and number of CDR3nt sequences obtained.**

|  |  |  | **Number of CDR3nt sequences^a^** | | | | | |
| --- | --- | --- | --- | --- | --- | --- | --- | --- |
| **Sample** | **Biological sex** | **Age** | **Unexpanded peripheral cells** | | | **Expanded peripheral T cells** | | |
|  |  |  | **PBMCs** | **CD4+** | **CD8+** | **T cells** | **CD4+** | **CD8+** |
|  |  |  | **TRB** | **TRB** | **TRB** | **TRB** | **TRB** | **TRB** |
| **P22-01** | M | 22 | 19368 | 43168 | 27802 | 10850 | 50741 | 26659 |
| **P22-02** | M | 28 | 22222 | 76592 | 12467 | 17393 | 49020 | 47908 |
| **P22-03** | F | 24 | 599 | 45368 | 67564 | 26779 | 36830 | 25522 |
| **P22-04** | F | 22 | 274 | 50328 | 16529 | 32177 | 48615 | 32710 |
| **P22-05** | M | 25 | 2588 | 73536 | 50577 | 10204 | 11075 | 5736 |
| **P22-06** | F | 25 | 4285 | 73532 | 45973 | 45973 | 60400 | 40717 |
| **Total** |  |  | 49336 | 362524 | 220912 | 143376 | 256681 | 179252 |

^a^Number of CD3nt sequences obtained by HTS in different samples analyzed.
